# Ogilvie’s Syndrome Following Herpes Zoster Infection: A Comprehensive Review of the Literature

**DOI:** 10.7759/cureus.74191

**Published:** 2024-11-21

**Authors:** Ioannis Loufopoulos, Unaiza Waheed, Elissavet Anestiadou, Antonios Kontos, Konstantinos S Kechagias, Konstantinos T Katsikas, Dimitrios Giannis, Georgios Geropoulos

**Affiliations:** 1 Department of Urology, Ipswich General Hospital, Ipswich, GBR; 2 Department of Surgery, Cambridge University Hospitals, Cambridge, GBR; 3 Fourth Surgical Department, George Papanikolaou General Hospital of Thessaloniki, Aristotle University of Thessaloniki, Thessaloniki, GRC; 4 Department of Obstetrics and Gynecology, Anaplasi Medical Rehabilitation Centre, Athens, GRC; 5 Department of Metabolism, Digestion, and Reproduction, Faculty of Medicine, Imperial College London, London, GBR; 6 Department of Nutrition and Dietetics, Royal Marsden Hospital, London, GBR; 7 Institute of Health System Science, Feinstein Institutes for Medical Research, New York, USA; 8 Department of General Surgery, Western General Hospital, Edinburgh, GBR

**Keywords:** herpes zoster virus, intestinal pseudo-obstruction, neuropathies, ogilvie's syndrome, varicella-zoster virus

## Abstract

This systematic review explores the association between herpes zoster (HZ) infection and Ogilvie’s syndrome (acute colonic pseudo-obstruction), evaluating how varicella-zoster virus (VZV) reactivation may contribute to autonomic dysfunction leading to intestinal obstruction. A comprehensive search was conducted in PubMed, Scopus, and Cochrane Library databases up to October 2024, in accordance with the Preferred Reporting Items for Systematic Reviews and Meta-Analyses (PRISMA) guidelines. Eligible studies included case reports, clinical images, and letters reporting Ogilvie’s syndrome secondary to HZ or VZV infection. After screening 219 publications and additional grey literature, 27 studies describing 28 cases met the inclusion criteria. Data were extracted on patient demographics, clinical manifestations, diagnostic methods, and outcomes. The quality of studies was assessed using the Joanna Briggs Institute critical appraisal checklist.

The results from 27 studies encompassing 28 patients with intestinal pseudo-obstruction secondary to VZV or HZ infection indicated a mean age of 60 years, predominantly affecting males (71.5%). Notably, 47.6% had underlying immunosuppressive conditions. The primary clinical manifestations included abdominal distention and severe constipation. Most patients (93%) exhibited a herpetiform rash, primarily in thoracic dermatomes. Symptoms of pseudo-obstruction often preceded the rash (58%), and imaging in the vast majority revealed colonic distension with no intra-abdominal pathology. Treatment focused on conservative management of both pseudo-obstruction and HZ symptoms, with 93% of patients achieving full recovery, while the mortality rate was identified at 7%.

The findings suggest that HZ-induced Ogilvie’s syndrome may be an underdiagnosed condition, requiring a high index of suspicion, particularly in immunocompromised patients. Early recognition and conservative treatment can prevent unnecessary surgical interventions. Further studies are needed to clarify the pathophysiological mechanisms linking VZV reactivation to colonic pseudo-obstruction.

## Introduction and background

Ogilvie’s syndrome due to herpes zoster (HZ) infection is a rare manifestation of varicella-zoster virus (VZV) reactivation. The onset of the rash of HZ and the symptoms of intestinal obstruction can occur at different time intervals posing a significant diagnostic challenge, accounting for avoidable surgical interventions.

HZ infection occurs in approximately 10% to 30% of individuals; it has been reported that 95% of the complications are sensory, most commonly in the form of acute pain syndromes and post-herpetic neuralgia [1]. Although motor injuries are less frequent, they have a more extensive presentation and can be either visceral or somatic (further classified into cranial and peripheral neuropathies).

Approximately 12% of HZ cases that occur on the head lead to peripheral neuropathies, with facial or oculomotor paralysis being the most frequently observed clinical symptom [2]. Visceral neuropathies may involve the bladder and cause cystitis and urinary retention. However, they can also lead to acute colonic pseudo-obstruction (ACPO), a phenomenon known as Ogilvie’s syndrome [3].

Bowel obstruction can be treated conservatively or with surgery. However, non-mechanical causes of bowel obstruction need to be carefully investigated prior to considering surgical intervention. The diagnosis of VZV-induced Ogilvie’s syndrome requires a high index of suspicion because visceral symptomatology may appear before the characteristic skin eruption [4,5]. The association of HZ and Ogilvie's syndrome is not common and has not been extensively studied in the literature. This systematic review aims to explore the ways by which VZV can induce Ogilvie's syndrome.

## Review

Materials & methods

This systematic review was conducted in accordance with the Preferred Reporting Items for Systematic Reviews and Meta-Analyses (PRISMA) guidelines [6]. The primary objective was to identify and analyze published reports of Ogilvie’s syndrome associated with HZ/VZV re-activation infection. The review protocol was agreed upon by all authors prior to initiation.

Eligibility and Search Strategy

Publications were deemed eligible for inclusion if they were original clinical studies or case reports focusing on Ogilvie’s syndrome (intestinal pseudo-obstruction) secondary to VZV/HZ infection. Inclusion criteria are summarized in all studies published in English, without restrictions on publication date. Due to the rarity of the clinical entity, letters to the editor and clinical images reporting original cases were also included in our analysis but not in the critical appraisal process. Exclusion criteria were non-English studies, review articles, and opinion pieces that lacked original case data. Studies whose full texts were not available online were also excluded from our analysis. Of note, studies describing cases where the intestinal symptoms were secondary to bowel contraction due to viral visceral involvement were also excluded.

Eligible studies were identified by searching PubMed (MEDLINE), Scopus, and Cochrane Library (end-of-search date: 10th October 2024) by two independent researchers (IL and JA). The search strategy was based on the following keywords: Ogilvie’s syndrome, herpes zoster virus (HZ), varicella-zoster virus (VZV), and intestinal pseudo-obstruction, combined with MeSH terms to capture all relevant studies. Any discrepancies in article inclusion were resolved by consensus or by the third senior author (GG). The reference lists and all previously published systematic reviews were thoroughly searched for missed studies eligible for inclusion based on the “snowball” methodology [7].

Data Extraction and Data Synthesis

A standardized, pre-piloted form was used for data tabulation and extraction. Two reviewers (IL and UW) extracted the data independently and any disagreements were identified and resolved by consensus. The following data was extracted from the included studies: (i) study characteristics (e.g., first author, year of publication, study design, sample size, and country); (ii) patient demographics (e.g., age, sex, and immune status); and (iii) clinical features (e.g., timing and nature of gastrointestinal and dermatologic symptoms, disease progress, and management strategies). Given the heterogeneity of the included studies and their design, a qualitative synthesis was only performed. The results were presented in a descriptive format, summarizing clinical characteristics, treatment modalities, and patient outcomes. Due to the limited number of cases and variability in data, a meta-analysis was not feasible.

Risk of Bias Assessment

Although no large clinical cohorts were identified, the methodological clinical assessment of the included studies/case reports was performed according to the Joanna Briggs Institute (JBI) critical appraisal checklist [8]. It is based on the quality of documentation of eight different elements, namely, patient demographics, medical history, history of current clinical status, diagnostic work-up and modalities, management/treatment of the clinical condition and subsequent outcomes, unanticipated or adverse events, and clear documentation of the aim of the reported case. Each section is scored with “Yes,” “No,” or “Unclear/not applicable” according to the availability and quality of the reported information. The included letters to the editors and clinical images, although reported in our analysis, were not assessed for bias as there is no formal tool for this purpose.

Results

Study Characteristics

**Figure 1:**
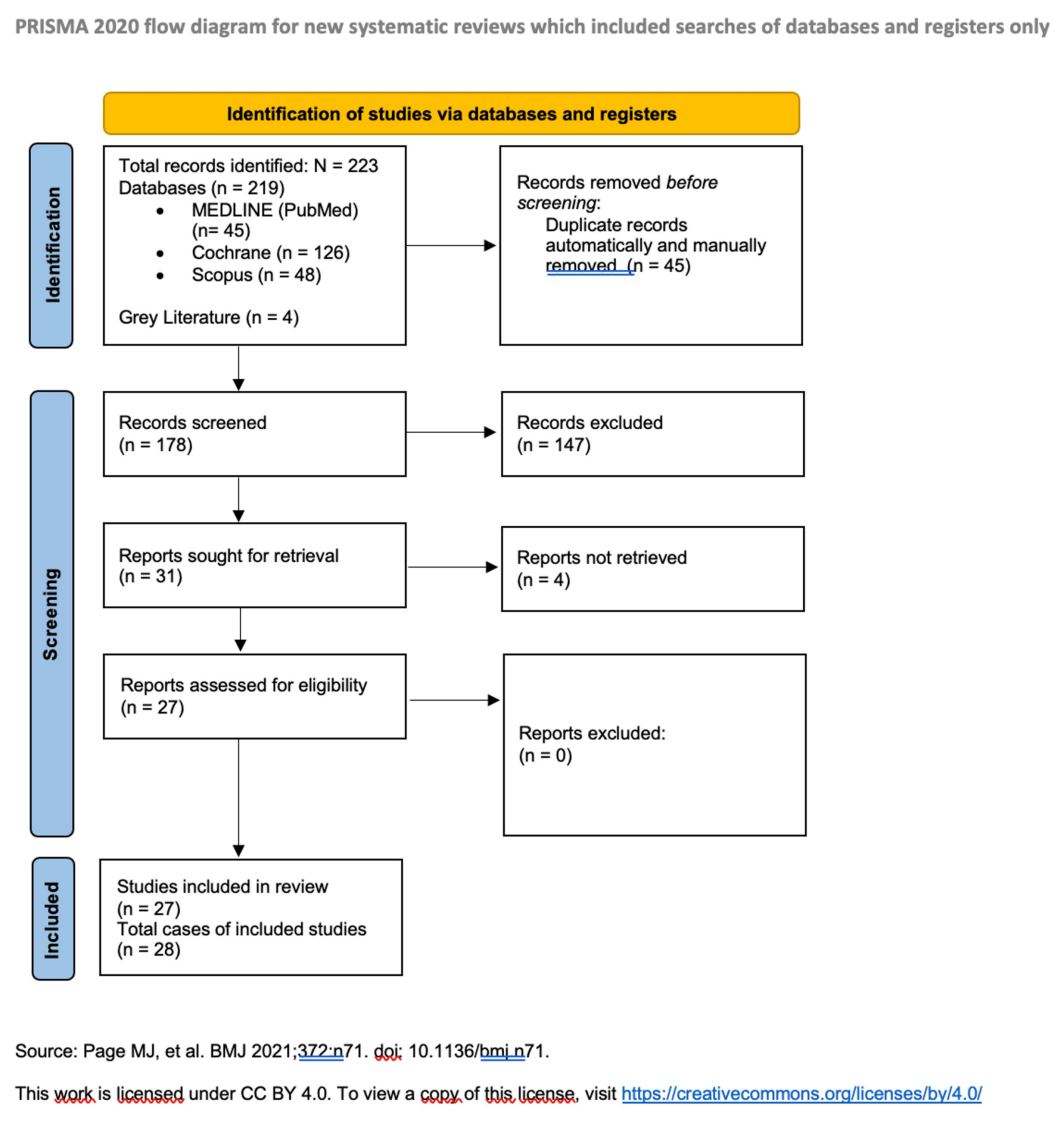
PRISMA flowchart. PRISMA: Preferred Reporting Items for Systematic Reviews and Meta-Analyses.

The initial literature search yielded 219 publications. Additional four studies were identified in the grey literature [9-12]. After screening for relevant titles and abstracts and removing duplicates, 31 full texts were reviewed for relevance. Ultimately, 27 studies [2-5,9-31] satisfied our eligibility criteria and were included in the final qualitative analysis (PRISMA flow chart, Figure 1 and Appendix 1). Published between 1977 and 2022, these studies consisted exclusively of case reports, letters to editors, and clinical images, with no large case series or cohorts identified. In more detail, 17 were published as case reports [2-5,11,12,15,16,19,21-25,28,30,31], five as clinical images [10,18,20,27,29], and five as letters to the editors [9,13,14,17,26]. All of them were original cases describing the clinical relevance of Ogilvie’s syndrome and concurrent VZV/HZ infection.

Patient Demographics

The 27 studies described 28 patients (Tribble et al. [12] reported two cases) with intestinal pseudo-obstruction secondary to VZV or HZ infection (Table 1). The mean age based on the available data, was 60 years (range = 5-84 years), with the vast majority being more than 50 years old (82%). Eight of them (28.5%) were females while 71.5% (20/28) were males. Geographic data were limited, and ethnicity associations could not be deduced. In 21 studies, past medical history was reported, with five patients being otherwise healthy, while 47.6% had underlying immunosuppressive conditions (e.g., 7/10 hematological malignancy, 4/10 post-transplant immunosuppression, and 2/10 HIV infection).

**Table 1:** Characteristics of included studies. HZ, herpes zoster; VZV, varicella-zoster virus; OS, Ogilvie’s syndrome; AXR, abdominal X-ray; CTAP, computed tomography of the abdomen and pelvis; MRI, magnetic resonance imaging; USS, ultrasonography; NAD, no abnormality detected; T, thoracic dermatome; NGT, nasogastric tube; NBM, nil by mouth; IVF, intravenous fluids; po, per os; PCR, polymerase chain reaction; ELISA, enzyme-linked immunosorbent assay; SIADH, syndrome of inappropriate antidiuretic hormone release; NA, not applicable; DLBCL, diffuse large B cell lymphoma; R-CHOP, rituximab, cyclophosphamide, doxorubicin, vincristine, prednisone; R-ESHAP, rituximab, etoposide, solu-medrone, high-dose cytarabine, cisplatin; ASCT, autologous stem-cell transplant; DM, diabetes mellitus; L, lumbar dermatome; GERD, gastroesophageal reflux disease; HTN, hypertension; IHD, ischemic heart disease; CKD, chronic kidney disease; S, sacral dermatome; CLL, chronic lymphocytic leukemia; ERBT, external beam radiation therapy; sc, subcutaneous; ABMT, autologous bone marrow transplant; DHAP, dexamethasone, high-dose cytarabine, cisplatin; Ig, immunoglobulin; HIV, human immunodeficiency virus; gpI, glycoprotein I; CNS, central nervous system; ARDS, acute respiratory distress syndrome; -ve, negative. * Epidural block: 1% lidocaine, 0.75% ropivacaine, betamethasone sodium phosphate, vitamin B12, and 0.9% normal saline.

First author/year	Study type	Age, gender	Comorbidities/immunological status	HZ/VZV symptoms	Location/dermatomes involved	Time interval between skin and intestinal symptoms	Abdominal imaging modalities and findings			Immunohistochemical (IH)/histopathology (HP)		Management	Outcome of pseudo-obstruction symptoms	Comments
							AXR/barium enema	CTAP/MRI/USS	Endoscopy/laparotomy	Smear from rash	Colonic biopsies			
Alpay et al. (1994) [13]	Letter to the editor	83, female	-	Vesicular zosteriform rash	Right T10	3 days after the obstruction symptoms	AXR: air-fluid levels, colonic dilatation with a cut-off point at splenic flexure	-	-	Tzanck test was positive for multinucleated giant cells	-	OS: NGT, NBM, IVF; HZ: po and topical acyclovir, po prednisolone	Improvement: 4 days; discharge: 7 days	-
Anaya-Prado et al. (2018) [2]	Case report	62, male	Nil	Vesicular zosteriform rash	Left T7-T10	24 hours after the obstruction symptoms	AXR: air-fluid levels, distended small bowel	CTAP: air-fluid levels, distended small bowel	-	-	-	OS: NGT, NBM, IVF; HZ: IV acyclovir	Resolution: 7 days; discharge: 8 days	-
Braude et al. (2015) [14]	Letter to the editor	64, female	Waldenstrom macroglobulinemia myelodysplasia secondary to rituximab, panobinostat, and fludarabine	Vesicular zosteriform rash	-	16 days after the obstruction symptoms	-	CTAP: non-obstructive pan-colonic dilatation (cecum up to 8.5 cm diameter), pericholecystic fluid, no gallstone	-	PCR: VZV DNA; ELISA: increased VZV IgG	PCR: VZV-DNA	OS: mechanical decompression with colonoscopy; VZV: IV acyclovir	Resolution: 3 weeks	Concurrent acalculous cholecystitis and SIADH
Caccese et al. (1985) [15]	Case report	75, male	Whipple’s procedure for common bile duct malignancy	Vesicular zosteriform rash	Right T9	5 days before the obstruction symptoms	AXR: large bowel dilatation with air cut-off distal to the splenic flexure; barium enema: same image, no obstruction	-	Colonoscopy: normal colon mucosa to caecum with no obstruction	-	-	OS: mechanical decompression with colonoscopy, NGT, NBM, IVF; HZ: NA	Improvement: 3 days; discharge: 5 days	-
Carrascosa et al. (2014) [16]	Case report	62, male	Stage IIIA DLBCL (R-CHOP, R-ESHAP, ASCT, consolidation chemotherapy with bendamustine 5 years ago), DM, previous HZ	Vesicular zosteriform rash	-	9 days after the obstruction symptoms	-	CTAP: marked colonic dilatation and possible narrowing at rectosigmoid junction	Colonoscopy: solitary sigmoid ulcer, no strictures	Rash biopsy: ulcer with epidermal necrosis and viral cytopathic effect; PCR: VZV DNA	Colonic biopsy: NAD; PCR: VZV DNA	OS: NA; VZV rash: po valacyclovir	Discharge: 19 days; follow-up in 9 months: complete resolution	-
Chung et al. (2017) [17]	Letter to the editor	75, female	Nil	Vesicular zosteriform rash	Right L4	-	AXR: diffuse colonic dilatation	CTAP: colonic dilatation, no obstruction	-	-	-	OS: NGT, NBM, IVF, bisacodyl, polyethylene glycol; HZ: po valaciclovir	Improvement: 3 days; resolution: 10 days	-
Edelman et al. (2010) [4]	Case report	84, male	GERD, HTN, hyperlipidemia, IHD, CKD, sigmoid colectomy for volvulus	Vesicular zosteriform rash	Right S2	4 days before the obstruction symptoms	-	CTAP with oral contrast: massive colonic distention, normal small bowel	Colonoscopy: colonic mucosa: features of ischemia due to bowel dilatation (cecum: 15 cm), no obstruction	-	-	OS: mechanical decompression with colonoscopy, laparotomy, and transverse colostomy; HZ: NA	Improvement: 5 days	Reason for initial admission: Mobitz type II atrioventricular block, requiring pacemaker
Haran et al. (2021) [18]	Clinical image	21, male	-	Vesicular zosteriform rash	Left L1-L2	9 days after the obstruction symptoms	AXR: NAD	CTAP: Distended cecum 9.3 cm without a transition point	-	-	-	Wet compressors, analgesia	Improvement: 4 days	-
Healy et al. (1998) [9]	Letter to the editor	84, male	CLL (chlorambucil)	Vesicular zosteriform rash	T11-T12	Simultaneous presentation	-	-	-	-	-	-	Resolution: 2 months; follow-up: 3 months later presented self-limiting posterior abdominal hernia	3 months follow-up: self-limiting abdominal wall hernia
Hosoe et al. (2011) [20]	Clinical image	58, male	-	Vesicular zosteriform rash	Left T8-T11	Simultaneous presentation	AXR: colonic dilatation, haustra only in proximal colon	CTAP and USS: no evidence of colonic spasm or obstruction	-	-	-	HZ: IV acyclovir, mosapride	-	-
Hong et al. (1998) [19]	Case report	69, male	-	Vesicular zosteriform rash	T12-L1	4 days before the obstruction symptoms	AXR: distended bowel loops, no physical impaction	-	-	Tzanck test was positive for multinucleated giant cells	-	OS: laxatives (lactulose); HZ: po famciclovir	Improvement: 48 hours	-
Johnson et al. (1977) [21]	Case report	51, male	Renal transplant (azathioprine and methylprednisolone)	Vesicular zosteriform rash	Right T12	2 days after the obstruction symptoms	AXR: gaseous distention of transverse colon, fluid levels in cecum and ascending colon; barium enema: NAD	-	Sigmoidoscopy: NAD	-	-	OS: NGT, NBM, IVF; HZ: analgesic, 40% idoxuridine in dimethyl sulphoxide	Resolution: 1 week	-
Jucgla et al. (1996) [22]	Case report	74 Female	Mycosis fungoides (ERBT)	Vesicular zosteriform rash	Right T10-L1	1 month before the obstruction symptoms	AXR: dilatation of ascending and transverse colon, minimal gas in the descending, presence of air-fluid levels; barium enema: NAD	-	Colonoscopy: NAD	-	-	OS: IVF, enemas; HZ: po acyclovir (1 month before)	Resolution: 2 weeks	-
Kesner et al. (1979) [23]	Case report	63, male	Nil	Vesicular zosteriform rash	Left L1-L2	10 days after the obstruction symptoms	AXR: gaseous distention of the colon proximally to the rectosigmoid junction, no fluid levels	-	Rectoscopy: NAD	-	-	OS: NGT, NBM, IVF	Resolution: 5 days	-
Lin et al. (2022) [24]	Case report	76, female	HTN	Vesicular zosteriform rash	T5-T10	5 days after the obstruction symptoms	AXR: dilatation of small bowel, no obvious obstruction	CTAP: dilatation of small bowel, no obvious obstruction; MRI: gallstones (misdiagnosed)	-	-	-	OS: NBM, enemas, epidural block therapy level T9-T10*; HZ: topical polymyxin B, red light irradiation, intramuscular acyclovir, sc B1 vit., po mecobalamin (no control of pain)	OS: resolution in 3-5 days; HZ: encrusted lesion in 3 days; complete recovery: 7 days	Epidural block treatment: complete resolution of symptoms; side effects: episode of hypotension treated with ephedrine hydrochloride
Maeda et al. (2007) [10]	Clinical image	66, male	HTN	Vesicular zosteriform rash	Left T11	4 days after the obstruction symptoms	AXR: NAD	-	-	-	-	-	-	-
Masood et al. (2015) [3]	Case report	35, male	Nil	Vesicular zosteriform rash	T8-T11	Simultaneous presentation	AXR: generalized gaseous distention of the colon with air up to the rectum	USS: similar to AXR	Colonoscopy: no obstruction	-	-	OS: NGT, NBM, IVF; HZ: IV acyclovir; discharge: po acyclovir and gabapentin or 10 days	OS: gradual resolution: 48-72 hours	-
Mechmet et al. (2012) [11]	Case report	80, male	DM	Vesicular zosteriform rash	Right T8-T11	6 days after the obstruction symptoms	AXR: NAD	-	Colonoscopy: NAD	-	-	HZ: po valacyclovir	Gradual resolution: 2 weeks	-
Nomdedeu et al. (1995) [25]	Case report	43, male	Stage IIIB Hodgkin’s lymphoma (multiple combinations of chemotherapy, ABMT- on cyclophosphamide and fractionated total body irradiation, prednisolone)	No skin symptoms	-	-	-	CTAP: dilatation of transverse colon and para-aortic lymphadenopathy	-	Autopsy: generalized herpetic infection with cytopathic changes: liver, esophagus, lung, skin	Mesenteric and coeliac plexuses: necrosis and intraneural hemorrhage; Colonic mucosa of splenic flexure: signs of ischemic necrosis; IH: no confirmation of HZ	-	Complicated: ARDS - death in 4 days	No IH confirmation due to poor quality of post-mortem samples
Precupanu et al. (2009) [26]	Letter to the editor	52, male	Stage II DLBCL (R-CHOP-21, DHAP, high-dose ERBT, ASCT)	Vesicular rash	Face, chest, arms, and legs	Simultaneous presentation	-	CTAP: focal thickening of the wall of the cecum and hepatic flexure, no obstruction	Sigmoidoscopy: colonic inflammation with small erythematous areas- intestinal viral lesions	PCR of smear from rash: VZV DNA; serology: anti-VZV IgG	-	HZ: po aciclovir for 8 weeks, IV Igs	Resolution of skin rash: 10 days; normal sigmoidoscopy: 50 days	Not received acyclovir prophylaxis after ASCT
Pui et al. (2001) [5]	Case report	34, male	HIV, Burkitt’s lymphoma	Diffuse vesicular zosteriform rash	-	Numerous days after the operation	-	CTAP: gaseous distention of the whole colon	Exploratory laparotomy: markedly inflamed terminal ileum and dilated right colon	-	Herpesviridae viral particles, Ulceration in the small bowel surrounded by hyperemic mucosa, submucosal/muscularis propria hemorrhage, and serosal fibrinous exudate; IH: VZV gpI staining of muscularis propria, myenteric plexus, lamina propria and media of blood vessels in the ulceration area	Laparotomy and partial ileocolectomy and ileostomy	Death: severe acute motor denervation and recurrent CNS lymphoma	Involvement of small bowel
Sousa et al. (2020) [27]	Clinical image	73, female	Bedridden, dementia, HTN, DM, previous stroke	No skin symptoms	-	-	AXR: megacolon	CTAP: marked distension of the sigmoid colon and rectum without anatomic lesion	Sigmoidoscopy: 2 triangular-shaped ulcers of approximately 20 mm diameter	-	Edema, vascular congestion, and lymphoplasmacytic infiltrate of the chorion with fibrin-leukocyte exudate and granulation tissue; PCR: VZV DNA	VZV: po acyclovir	Follow-up sigmoidoscopy: complete resolution	No skin manifestations
Supangat et al. (2022) [28]	Case report	5, female	-	Vesicular rash	Diffuse in the whole body	Five days after the obstruction symptoms	AXR: stepladder pattern of colon with air-fluid levels	-	Exploratory laparotomy: intussusception and perforation of the ileocecal junction with enlargement of local lymph node	-	-	OS: rectal tube decompression; VZV: topical acyclovir; intussusception: exploratory laparotomy and ileostomy	Complication: intussusception; discharge: 16 days	Early complication: intussusception
Tribble et al. (1993) [12]	Case reports	60, male	Waldenstrom’s macroglobulinemia (multiagent chemotherapy)	Vesicular zosteriform rash	Left T8-T9	5 days after the obstruction symptoms	AXR: diffuse distention of the colon with minimal gas in the small bowel and no air in the rectum	-	Colonoscopy: no obstruction	Tzanck test was positive for multinucleated giant cells	-	OS: colonoscopy decompression; HZ: IV acyclovir	Gradual resolution	-
		35, male	HIV-1, Kaposi's sarcoma, subarachnoid hemorrhage. (zidovudine, fluconazole, trimethoprim-sulfamethoxazole)	Vesicular zosteriform rash	Right T6	9 days after the obstruction symptoms	AXR: dilatation of the transverse colon with minimal gas in the descending colon	-	Colonoscopy: dilated segment, normal mucosa, no obstruction	Tzanck test was positive for multinucleated giant cells. Cultures for VZV and herpes simplex: (-ve)	-	OS: NGT, NBM, IVF; HZ: IV acyclovir	Gradual resolution	Anti-retroviral treatment missed for 6 days before symptoms
Tsukita et al. (2019) [29]	Clinical image	71, male	-	Vesicular zosteriform rash	Left T11-T12	Simultaneous presentation	AXR: marked diffuse colonic dilatation	CTAP: no obstruction	-	-	-	HZ: IV acyclovir	Complication: abdominal wall pseudo-hernia. Gradual resolution	Complication: abdominal wall pseudo-hernia
Walsh et al. (1982) [30]	Case report	68, male	Nil	Typical vesicular rash in the healing stage	-	2 days before the obstruction symptoms	AXR: distended colon up to splenic flexure, no cut-off point	-	Exploratory laparotomy: distended caecum, ascending and transverse colon, collapsed descending colon	-	-	Laparotomy and transverse colostomy in view of misdiagnosed splenic flexure tumor	Gradual resolution	-
Zhou et al. (2012) [31]	Case report	59, female	-	Vesicular zosteriform rash	Left abdomen and waist	-	AXR: two air-fluid levels	-	-	-	-	OS: NBM, IVF, enema, moxifloxacin; HZ: valacyclovir, vit. B12, analgesia for 3 weeks	OS improvement: 5 days; HZ resolution: 15 days; in 1 month: constipation and pain resolved, persistence of mild left distention	-

Clinical Manifestation and Diagnostic Workup

The predominant symptoms included abdominal distention (20/28), accompanied by severe to absolute constipation (21/28). Diffuse or localized pain of colicky character was also notable in the vast majority of the patients. Four of them had symptoms of complete bowel obstruction with concomitant vomiting. On clinical examination and auscultation of the abdomen, bowel sounds were hypoactive or hyperactive in a ratio of 2:1. Percussion demonstrated tympanic, and digital rectal examination was normal in the vast majority. In regards to viral manifestations, 26 (93%) of the patients had vesicular/papular herpetiform rash with only two patients (7%) lacking skin symptoms despite a confirmed histopathological diagnosis of Herpesviridae DNA. Dermal distribution-wise, the rash involved predominantly the thoracic dermatomes in 15 cases, the lumbar ones in five, and the sacral in one case. Twenty-five studies reported the time interval between the onset of skin and intestinal manifestations. In 58% of cases, pseudo-obstruction symptoms preceded the skin rash (range: 1-16 days) while in equal percentages (21%) it followed (range: two days to one month) or it was simultaneous to the onset of the vesicular rash.

Different imaging modalities, including abdominal X-rays, barium enemas, and CT scans were used for the investigation of the intestinal symptoms, typically revealing colonic distension, with air-fluid levels and no underlying intra-abdominal pathology. In 11 cases, an endoscopic assessment of the bowel/rectum (colonoscopy/sigmoidoscopy) was performed with two identifying colonic mucosa inflammation and signs of ischemia and two demonstrating superficial ulcers but free of any malignant pathology. The rest seven were completely normal. Histopathological and immunohistochemical confirmations of Ogilvie’s syndrome with VZV infection were present in nine patients, with positive Tzanck tests in four cases and polymerase chain reaction (PCR)-confirmed VZV DNA in four others (either in colonic biopsy tissues or rash smear samples). However, one patient who was Tzanck test positive had negative PCR for VZV. Interestingly, the micro-vascularity of the colon was studied and identified to be affected in 3/28 (11%) patients.

Management and Outcomes

Treatment strategies varied but primarily involved conservative management, including bowel rest, nasogastric decompression, laxatives and enemas, and adequate analgesia. Intravenous or oral antiviral therapy, such as acyclovir or valacyclovir, was constituting the main way of management of the skin manifestations with steroids used in select cases. Severe cases required colonoscopic decompression (14%) or epidural block therapy at the levels of the affected dermatomes (one case reported). Three cases were mistakenly diagnosed as mechanical bowel obstruction and underwent diagnostic laparotomy with stoma formation. One patient developed intussusception and bowel perforation of the ileocecal junction secondary to herpes-induced reactive enlarged lymph node, as an early complication of Ogilvie’s syndrome. She was treated with laparotomy and end-ileostomy. Of note, two cases were complicated with self-resolving abdominal wall pseudo-hernias. Ultimately, the majority of the cases (93%) achieved full recovery, with a mortality rate of 7%, attributed to acute respiratory distress syndrome and primary lymphoma recurrence.

Results of Risk of Bias Assessment

Quality assessment, summarized in Table 2, indicated that most studies met essential criteria, with 11 studies classified as low risk (high score in six or more available domains) [2-4,12,15,16,21,23-25,28]. Six studies scored low in over three elements, often due to limited data availability [5,11,19,22,30,31]. Domain 8 showed lower scores in nearly half of the studies (7/17).

**Table 2:** Quality assessment of the included case reports. Q1: Were the patient’s demographic characteristics clearly described? Q2: Was the patient’s history clearly described and presented as a timeline? Q3: Was the current clinical condition of the patient on presentation clearly described? Q4: Were diagnostic tests or methods and the results clearly described? Q5: Was the intervention(s) or treatment procedure(s) clearly described? Q6: Was the post-intervention clinical condition clearly described? Q7: Were adverse events (harms) or unanticipated events identified and described? Q8: Does the case report provide takeaway lessons? "+": Yes; "-" = No; NA = Not applicable.

References	Q1	Q2	Q3	Q4	Q5	Q6	Q7	Q8
Anaya-Prado et al. (2018) [2]	-	-	+	+	+	+	NA	+
Caccese et al. (1985) [15]	+	+	+	+	+	+	NA	+
Carrascosa et al. (2014) [16]	+	+	+	+	+	+	NA	+
Edelman et al. (2010) [4]	+	+	+	+	+	+	+	+
Hong et al. (1998) [19]	-	-	+	-	-	-	NA	-
Johnson et al. (1977) [21]	+	+	+	+	+	+	NA	-
Jucgla et al. (1996) [22]	+	+	+	+	-	-	NA	-
Kesner et al. (1979) [23]	+	+	+	+	-	+	NA	-
Lin et al. (2022) [24]	+	+	+	+	+	+	NA	+
Masood et al. (2015) [3]	+	+	+	+	+	+	NA	+
Mechmet et al. (2012) [11]	+	-	+	-	-	-	NA	-
Nomdedeu et al. (1995) [25]	+	+	+	+	+	+	+	+
Pui et al. (2001) [5]	+	+	-	+	-	+	+	-
Supangat et al. (2022) [28]	+	-	+	+	+	+	+	+
Tribble et al. (1993) [12]	+	+	+	+	+	+	NA	+
Walsh et al. (1982) [30]	+	+	-	-	-	-	NA	+
Zhou et al. (2012) [31]	+	-	-	-	+	+	NA	-

Discussion

The relationship between VZV infection and the development of Ogilvie’s syndrome remains a rare but clinically significant phenomenon. The literature suggests several mechanisms by which HZ infection may lead to Ogilvie's syndrome. Autonomic dysfunction secondary to viral reactivation is the most commonly cited cause. However, there is variability in how these cases present, with some authors suggesting that age and underlying conditions may exacerbate the risk, while others highlight the role of immunosuppression as a critical factor. This review explores the natural history and the potential mechanisms by which VZV may induce Ogilvie's syndrome, focusing on the pathophysiological effects of viral reactivation on autonomic nervous system function, while highlighting key gaps and inconsistencies within the existing literature.

Clinically, Ogilvie's syndrome resembles mechanical colonic obstruction presenting with abdominal pain, distention, and constipation, with or without nausea and vomiting [32]. The pathophysiology of Ogilvie’s syndrome is obscure and numerous theories have been proposed. The most prevalent one correlates Ogilvie's syndrome with autonomic denervation, suggesting that the disease occurs due to autonomic nervous system dysregulation, including sympathetic and/or parasympathetic dysfunction [33].

Primary infection with VZV typically manifests as a diffuse, pruritic rash accompanied by viremia, while reactivation of latent VZV results in HZ, commonly known as shingles [34]. VZV is a neurotropic alphaherpesvirus that enters a state of latency within neural tissues following the initial infection. Specifically, the virus has been detected in the dorsal root ganglia, cranial nerve ganglia, and autonomic ganglia within the enteric nervous system, where it remains dormant throughout a person's lifetime. Reactivation of VZV is often triggered by factors such as advanced age, immunosuppression (whether disease-related or iatrogenic), stress, malnutrition, and hormonal changes like menstruation [35].

**Figure 2:**
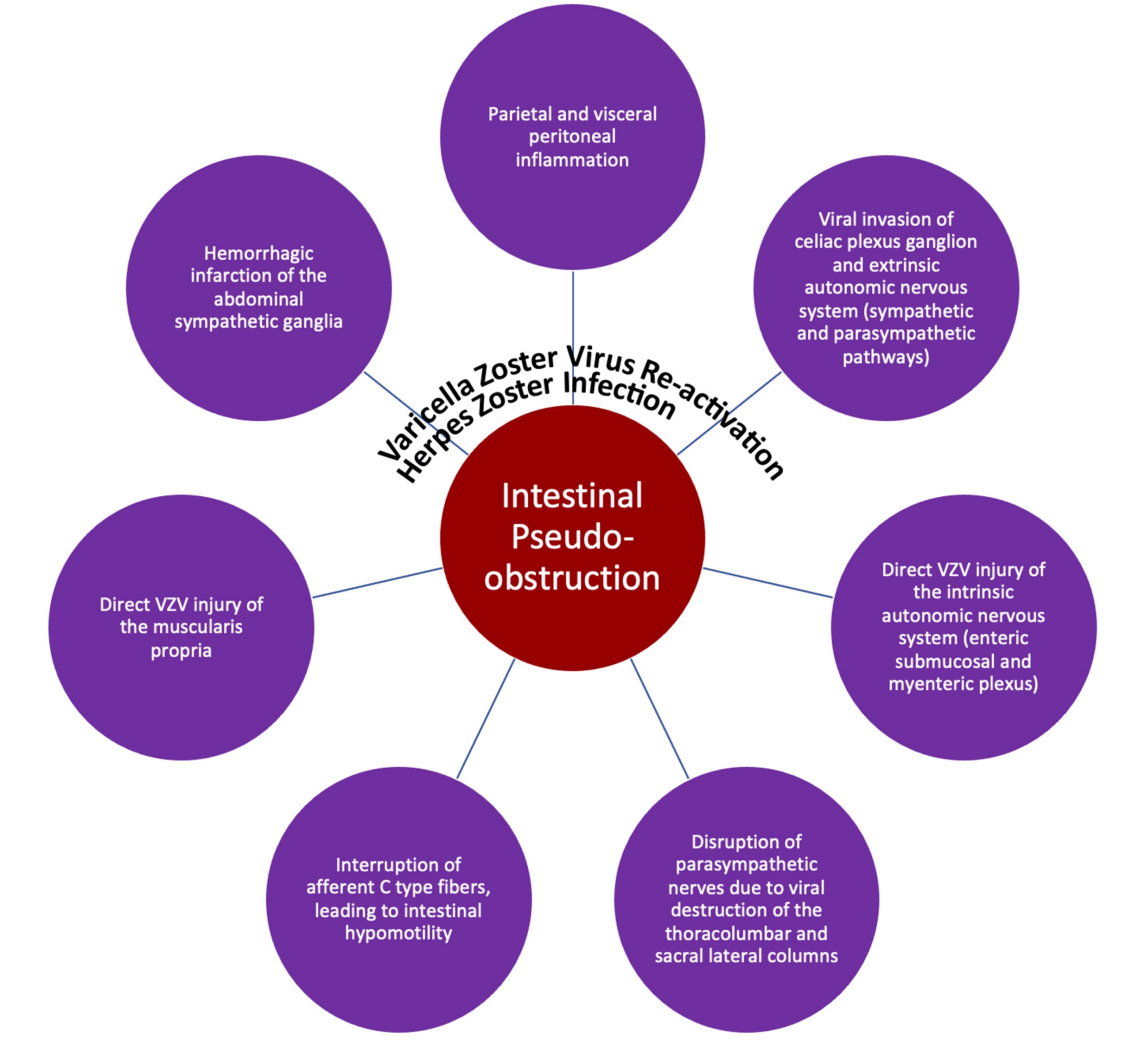
Summary of pathophysiological mechanisms correlating herpes zoster infection and Ogilvie’s syndrome. The image was prepared by the authors.

Beyond its well-known skin manifestations, VZV is associated with various somatic and visceral motor neuropathies, affecting approximately 5% of patients [2]. In the abdomen, VZV has been detected in structures including the dorsal root ganglia, spinal cord, adrenal glands, and the enteric nervous system (ENS) ganglia. VZV may enter the ENS in two main ways: by traveling with T lymphocytes during the acute infection, which allows the virus to establish latency in the ENS, or through retrograde axonal transport, moving from infected neurons in the dorsal root ganglia via epidermal projections [3]. The mechanisms linking these pathophysiological processes to the bowel distention characteristic of Ogilvie’s syndrome are varied and complex (Figure 2).

VZV's impact on the autonomic nervous system is central to the development of colonic pseudo-obstruction, with neurogenic involvement being a primary mechanism. The virus may invade the extrinsic autonomic ganglia, disrupting autonomic signaling pathways that regulate colonic motility. This mechanism is suggested by Pui et al. [5], who posit VZV invasion of the celiac plexus and sympathetic and parasympathetic pathways. Additionally, Tribble et al.’s [12] theory on the destruction of thoracolumbar and sacral lateral columns by VZV outlines how damage to these regions disrupts autonomic balance, affecting bowel peristalsis and further impairing motility. Autonomic dysregulation due to VZV reactivation can create an imbalance in sympathetic and parasympathetic signaling. Hosoe et al.’s [20] theory on the interruption of afferent C-type fibers emphasizes how this disruption impairs colonic motility by creating a hypomotile state, contributing to pseudo-obstruction. Direct viral injury and inflammation also contribute significantly to colonic dysfunction. Pui et al.’s [5] theory of direct VZV injury to the intrinsic enteric plexuses (submucosal and myenteric) describes how the virus disrupts enteric nerve function, impairing motility. Furthermore, Nomdedéu et al.’s [25] theory on inflammation of the parietal and visceral peritoneum suggests that VZV-induced inflammation can exacerbate nerve dysfunction in the GI tract, compounding the pseudo-obstruction. Vascular and ischemic factors may additionally play a role. VZV infection can initiate a pro-inflammatory cascade that impairs blood flow, leading to ischemia in the autonomic ganglia. Nomdedéu et al.’s [25] theory of hemorrhagic infarction in the abdominal sympathetic ganglia describes how such ischemia may worsen autonomic dysfunction, disrupting motility further. Finally, muscular and nervous system involvement, supported by histopathological findings, highlights direct injury to the bowel wall. Pui et al.’s [5] theory of VZV injury to the muscularis propria illustrates how this damage compromises the structural integrity and motility of the bowel, contributing directly to functional obstruction.

Ogilvie’s syndrome has been linked to a range of viral infections, including severe acute respiratory syndrome-coronavirus-2 (SARS-CoV-2) [36], cytomegalovirus (CMV), John Cunningham virus (JCV), herpes simplex virus (HSV), Epstein-Barr virus (EBV), VZV, and flaviviruses [37]. Additionally, cases of Ogilvie’s syndrome have been reported in patients with acquired immunodeficiency syndrome (AIDS) due to human immunodeficiency virus (HIV) infection [38]. These associations reinforce the idea that VZV and other neurotropic viruses could be underlying triggers for the development of Ogilvie’s syndrome, particularly through the mechanism of autonomic dysfunction.

This systematic review synthesizes available case reports and small studies on the clinical presentation, diagnosis, and management of Ogilvie’s syndrome in patients with concurrent HZ or VZV reactivation. Our review identified 27 studies describing 28 cases, revealing important insights into the demographic profile, clinical features, diagnostic approaches, and outcomes associated with this rare complication. The majority of patients were older adults, with a mean age of 60 years and 82% over 50, consistent with the age-related increased susceptibility to HZ reactivation due to declining cellular immunity. Immunosuppression was present in nearly half of the cases, underscoring the association between compromised immunity and the development of HZ-related complications, including intestinal pseudo-obstruction. This finding supports prior research that identifies immunocompromised patients, particularly those with hematologic malignancies or undergoing chemotherapy, as having an elevated risk of severe and atypical manifestations of HZ.

Clinically, the patients presented with symptoms characteristic of Ogilvie’s syndrome, including abdominal distention, constipation, and colicky abdominal pain. Notably, 93% of patients had a characteristic herpetiform rash, mainly along thoracic dermatomes, although in some cases, the gastrointestinal symptoms preceded the rash by up to 16 days. This temporal variation in the onset of pseudo-obstruction relative to rash presentation complicates diagnosis and highlights the need for heightened clinical suspicion in HZ or VZV patients presenting with bowel symptoms.

Imaging studies primarily revealed colonic distension without evidence of obstructive pathology, consistent with Ogilvie’s syndrome. In cases where endoscopy was performed, findings were often nonspecific, with only a minority showing signs of colonic inflammation or ischemia. Positive identification of VZV or HZ infection via histopathology or immunohistochemical testing in bowel biopsies or rash samples was reported in a few cases, including Tzanck testing and PCR for VZV DNA, albeit inconsistently. This underscores the diagnostic complexity and suggests that a comprehensive work-up, including both imaging and viral testing, may improve diagnostic accuracy for HZ-induced Ogilvie’s syndrome.

Management was predominantly conservative, with antiviral therapy and bowel rest as the mainstays of treatment. More severe cases required decompressive interventions, either through colonoscopy or, in three cases, diagnostic laparotomy. Outcomes were generally favorable, with 93% of patients achieving complete resolution, although two cases resulted in mortality due to complications related to their underlying conditions. This highlights the importance of accurate diagnosis and prompt antiviral treatment to prevent further complications in immunosuppressed patients.

In terms of limitations, the lack of large cohort studies restricts the generalizability of these findings, as this review relied exclusively on case reports, letters to the editors, and clinical images of low levels of evidence and quality (Table 2). Further research with larger samples is needed to confirm the observed associations and to clarify the pathophysiological mechanisms underlying Ogilvie’s syndrome in HZ patients. Additionally, standardized diagnostic criteria for identifying VZV in gastrointestinal tissue samples could aid in consistent diagnosis and reporting.

## Conclusions

In summary, our findings suggest that Ogilvie’s syndrome, though rare, is a clinically significant complication of HZ infection, particularly in older and immunocompromised patients. The unclear relationship between HZ symptoms and pseudo-obstruction highlights the need for heightened awareness among clinicians. Prompt recognition and treatment are crucial for favorable outcomes, and future studies should aim to elucidate the pathogenesis and optimal management strategies for this unique manifestation of VZV infection.

## Appendices

Appendix 1: Reasons for exclusion

• Wrong population (n = 128)

• Wrong publication type (n = 30)

• Wrong study design (n = 18)

• Non-English (n = 3)

* 32 studies had more than one reason for exclusion.
